# Firewalls Prevent Systemic Dissemination of Vectors Derived from Human Adenovirus Type 5 and Suppress Production of Transgene-Encoded Antigen in a Murine Model of Oral Vaccination

**DOI:** 10.3389/fcimb.2018.00006

**Published:** 2018-01-25

**Authors:** Julien Revaud, Yves Unterfinger, Nicolas Rol, Muhammad Suleman, Julia Shaw, Sandra Galea, Françoise Gavard, Sandrine A. Lacour, Muriel Coulpier, Nicolas Versillé, Menzo Havenga, Bernard Klonjkowski, Gina Zanella, Stéphane Biacchesi, Nathalie Cordonnier, Blaise Corthésy, Juliette Ben Arous, Jennifer P. Richardson

**Affiliations:** ^1^UMR Virologie INRA, Ecole Nationale Vétérinaire d'Alfort, ANSES, Université Paris-Est, Maisons-Alfort, France; ^2^SEPPIC Paris La Défense, Paris, France; ^3^R&D Laboratory, Division of Immunology and Allergy, Centre des Laboratoires d'Epalinges, Centre Hospitalier Universitaire Vaudois, Lausanne, Switzerland; ^4^Batavia Biosciences B.V., Leiden, Netherlands; ^5^Anses, Epidemiology Unit, Laboratoire de Santé Animale, Université Paris-Est, Maisons-Alfort, France; ^6^VIM, INRA, Université Paris-Saclay, Jouy-en-Josas, France

**Keywords:** oral vaccination, viral vector, adenovirus, M cell, Peyer's patch

## Abstract

To define the bottlenecks that restrict antigen expression after oral administration of viral-vectored vaccines, we tracked vectors derived from the human adenovirus type 5 at whole body, tissue, and cellular scales throughout the digestive tract in a murine model of oral delivery. After intragastric administration of vectors encoding firefly luciferase or a model antigen, detectable levels of transgene-encoded protein or mRNA were confined to the intestine, and restricted to delimited anatomical zones. Expression of luciferase in the form of multiple small bioluminescent foci in the distal ileum, cecum, and proximal colon suggested multiple crossing points. Many foci were unassociated with visible Peyer's patches, implying that transduced cells lay in proximity to villous rather than follicle-associated epithelium, as supported by detection of transgene-encoded antigen in villous epithelial cells. Transgene-encoded mRNA but not protein was readily detected in Peyer's patches, suggesting that post-transcriptional regulation of viral gene expression might limit expression of transgene-encoded antigen in this tissue. To characterize the pathways by which the vector crossed the intestinal epithelium and encountered sentinel cells, a fluorescent-labeled vector was administered to mice by the intragastric route or inoculated into ligated intestinal loops comprising a Peyer's patch. The vector adhered selectively to microfold cells in the follicle-associated epithelium, and, after translocation to the subepithelial dome region, was captured by phagocytes that expressed CD11c and lysozyme. In conclusion, although a large number of crossing events took place throughout the intestine within and without Peyer's patches, multiple firewalls prevented systemic dissemination of vector and suppressed production of transgene-encoded protein in Peyer's patches.

## Introduction

Among the non-replicative viral vectors that have been explored as vaccine carriers, those derived from human adenovirus type 5 (HAdV-5) have proven particularly effective in eliciting immune responses against transgene-encoded antigen (Shiver et al., 2002). Whereas this observation strictly concerns parenteral delivery routes, orally-delivered vaccines are also sought, owing to their ease of delivery in humans and other species and their potential to elicit mucosal immunity. In this capacity, however, non-replicative HAdV5-based vaccines have proven less effective. Though oral administration of HAdV-5-based vaccines has given rise to systemic immune responses against the transgene product in multiple animal species, including rodents (Xiang Z. Q. et al., 2003) and primates (Mercier et al., 2007), as well as in wild and domestic species, such as foxes, dogs, and pigs (Hammond et al., 2001, 2003; Tuboly and Nagy, 2001; Vos et al., 2001; Zhang et al., 2008), they have been considered weak, especially in large animals (Lubeck et al., 1989; Lin et al., 2007). Moreover, when different delivery routes have been compared in the same trial, higher doses have generally been required to afford protection by the oral route (Ertl, 2005).

The bottlenecks to efficient induction of immune responses after oral delivery of HAdV-5-based vaccines remain ill-defined. Instability in the gastric environment diminishes the dose of viable vector that reaches the small intestine, but cannot be the only limiting factor, since even when the stomach has been bypassed by enteric administration in sheep or endoscopic deposition in the small intestine of dogs, adaptive immune responses remained weak (Mutwiri et al., 2000) or undetectable (Vos et al., 2001), respectively. A high affinity receptor for HAdV-5, the coxsackievirus and adenovirus receptor (CAR) (Bergelson et al., 1997), and major internalization co-receptors, the αv integrins (Wickham et al., 1993), have been implicated in transduction of highly permissive cell lines. These molecules, however, are only weakly expressed at the apical surface of differentiated intestinal epithelial cells, presumably limiting their transduction by luminal HAdV-5 particles (Hamilton et al., 1997; Walter et al., 1997; Croyle et al., 1998a,b; Lecollinet et al., 2006; Kesisoglou et al., 2010). Even once the mucosal barrier has been overcome, the tolerogenic context of the intestinal milieu may represent an additional bottleneck, and indeed systemic IgG responses elicited against a transgene-encoded antigen were substantially enhanced when an intragastrically delivered HAdV-5-derived vector encoded a Toll-like receptor 3 ligand (Scallan et al., 2013).

The transfer of vaccinal antigen to professional antigen-presenting cells, and notably to dendritic cells (DCs), has emerged as a determinant factor in vaccine efficacy (Steinman, 2012). Such transfer is particularly critical in oral vaccine delivery, since the barrier function of the gastrointestinal mucosa restricts translocation of luminal antigen across the epithelium, and thus its recovery by underlying DCs. Nevertheless, once introduced into the gut, antigen may be transported across both follicle-associated epithelium (FAE) and the villous epithelium (for a review see Schulz and Pabst, 2013). The FAE overlies clusters of lymphoid follicles, called Peyer's patches, and is distinguished by the presence of specialized transcytotic cells, called microfold (M) cells. M cells actively transport particulate luminal antigen toward the subepithelial dome (SED) region (Owen, 1999; Neutra et al., 2001), where a myriad of mononuclear phagocytes lie in wait. In villous epithelium, immunoglobulin-bound antigen may be transported from the lumen to the lamina propria by antigen-shuttling receptors of epithelial cells (Yoshida et al., 2004) or, as proposed in a recent study (McDole et al., 2012), by goblet cells. Moreover, dendritic extensions of phagocytic cells can penetrate the villous epithelium by the paracellular pathway and capture particulate antigen at the luminal surface of epithelial cells or in the lumen (Farache et al., 2013). For several enteric viruses, including reovirus (Wolf et al., 1981) and norovirus (Gonzalez-Hernandez et al., 2014; Kolawole et al., 2015), Peyer's patches—and in some instances, M cells—have explicitly been shown to play a major role in entry of luminal virus, but this possibility remains to be addressed for HAdV5.

In order to improve the immunogenicity of orally-delivered HAdV-5-based vectors, a better understanding of their fate in the intestinal milieu is a prerequisite. To provide a global vision of transgene expression in the intestine, bioluminescence imaging of the entire digestive tract was performed in mice after intragastric administration of a HAdV-5-based vector encoding firefly luciferase. In complementary experiments, quantitative RT-PCR was performed in the intestine and in peripheral tissues after intragastric delivery of vector. Finally, in order to characterize the pathways by which the vector traverses the intestinal epithelium and is captured by sentinel cells, a fluorescent-labeled vector was administered to mice by the intragastric route or inoculated into ligated intestinal loops comprising a Peyer's patch. These studies have allowed us to trace the route taken by intragastrically delivered HAdV-5-based vectors from the lumen of the intestine to gut-associated lymphoid tissue.

## Materials and methods

### Mice

Animal experimentation was conducted in compliance with European and institutional guidelines and approved by the local ethics committee (ComEth Anses/ENVA/UPEC: 20/12/12-26B). Female C57Bl/6 and BALB/c mice were purchased from Charles River Laboratories and housed under specific pathogen-free conditions. Mice were 8 weeks of age at the beginning of each protocol.

### Cell culture

The human embryonic kidney cell line HEK-293 (Graham et al., 1977), used for propagation and titration of HAdV-5-based vectors, was cultivated in Dulbecco's modified Eagle's medium containing 4 mM L-alanyl-L-glutamine dipeptide (GlutaMax) and supplemented with 1 mM pyruvate, 10% heat-inactivated fetal calf serum (FCS) (Eurobio), 100 IU/ml penicillin and 100 μg/ml streptomycin (complete DMEM medium). Cells were maintained at 37°C in the presence of 5% CO_2_.

### Adenovirus-based vectors

Vectors were derived from E1- and E3-deleted HAdV-5; Ad5-*ova* (Suleman et al., 2011), Ad5-*luc* (Havenga et al., 2001), and Ad5-*gfp* (the kind gift of Michael Barry; Parrott et al., 2003) expressed chicken ovalbumin (ova), firefly luciferase (luc), and green fluorescent protein (GFP), respectively, under the control of the intermediate-early cytomegalovirus (IE-CMV) promoter (full-length for Ad5-*ova* and -*gfp* and minimal for Ad5-*luc*). These were amplified in HEK-293 cells and purified by two rounds of CsCl gradient ultracentrifugation. Vectors were desalted by gel filtration on columns (Disposable PD-10 Desalting Column with Sephadex G-25 resin, GE Healthcare) equilibrated with phosphate-buffered saline (PBS). Following addition of glycerol (10%, v/v), vectors were aliquoted and stored at −80°C. The concentration of viral physical particles was determined by measurement of absorbance at 260 nm, after dilution of the viral suspension in sodium dodecyl sulfate (1% final concentration) and incubation at 100°C (Sweeney and Hennessey, 2002). Infectious titers were determined by endpoint dilution in HEK-293 cells (Reed and Muench, 1938). The presence of a cytopathic effect was assessed under light microscopy and titers were expressed as median tissue culture infective doses (TCID_50_).

### Immunization of mice with Ad5-ova

The ova-specific humoral response elicited after administration of the Ad5-ova vector by intragastric and intramuscular delivery routes was assessed by ELISA. Groups of five C57Bl/6 mice were immunized with 5 × 10^6^ or 5 × 10^8^ TCID_50_ of Ad5-*ova* in 100 μl of PBS at days 0 and 28. As a positive control, a third group received 300 μg of purified ova (Grade 5, Sigma-Aldrich), along with 10 μg of cholera toxin (Sigma-Aldrich), in 100 μl of PBS at days 0, 7, 14, and 28 by the intragastric route. A feeding needle was used for intragastric delivery, and mice were fasted for approximately 12 h prior to administration. For intramuscular delivery 50 μl of inoculum was inoculated into each hind limb for both prime and boost. Blood was collected 1 week prior to immunization and then at 2-week intervals from day 0 on, for preparation of serum.

Each well of 96-well microplates (Maxisorp, Nunc) was coated with 2 μg of ova in 100 μl of carbonate-bicarbonate buffer (0.05 M, pH 9.6) and incubated overnight at 4°C. Plates were then washed with PBS containing 0.05% Tween 20 (PBS-Tween), as for all subsequent washes. Plates were blocked by incubation with 200 μl of PBS containing 1% bovine serum albumin (BSA) and incubated for 2 h at room temperature. After washing, 100 μl volumes of dilutions of mouse sera prepared in PBS-Tween containing 1% BSA (PBS-Tween-BSA) were added and incubated for 2 h at room temperature, as were serial dilutions of a murine monoclonal antibody (mAb) recognizing ova (Sigma-Aldrich, clone OVA-14). After washing, 100 μl volumes of a 1/8000 dilution of horseradish peroxidase-conjugated goat F(ab')2 anti-mouse pan-Ig (SouthernBiotech) in PBS-Tween-BSA were added and incubated for 1 h at room temperature. After washing, 100 μl volumes of 3,3′,5,5′-tetramethylbenzidine substrate (Invitrogen) were added and absorbance was measured 15 min later at 405 nm after addition of 100 μl of 1 M HCl. A standard curve was constructed in which absorbance was plotted as a function of the concentration of anti-ova mAb. Anti-ova titers in mouse sera were expressed as the concentration of anti-ova antibody, by reference to the standard curve.

### Evaluation of transgene expression by bioluminescence

Mice were fasted for ~12 h prior to experiments. Ad5-*luc* was adjusted to 2.5 × 10^9^ TCID_50_/ml in PBS. Fasted mice were anesthetized with isoflurane prior to intragastric administration of 200 μl of Ad5-*luc* (5 × 10^8^ TCID_50_) by means of a feeding needle. Twenty-four hours later, the mice were anesthetized by intraperitoneal injection of ketamine (100 mg/kg) and xylazine (10 mg/kg). A volume of 100 μl of d-luciferin (30 mg/ml in PBS) was then administered by intraperitoneal injection (2 sites of injection), so as to administer 150 mg/kg, or 3 mg for a mouse weighing 20 g. After 5 min, bioluminescence imaging was performed on live mice (BALB/c only) and—following euthanasia and rapid dissection of mice—on the entire intestine by using the IVIS 200 imaging system (PerkinElmer). Living Image software (version 4.0, PerkinElmer) was used both to acquire bioluminescent and photographic images and perform analyses. Bioluminescent images were acquired for 1 min with f/stop and binning settings of 1 and medium, respectively, from which background bioluminescence was subtracted. For analysis of bioluminescence, image data were displayed as photons, and regions of interest (bioluminescent foci) were drawn using the automatic function, such that only regions in which the number of pixels exceeded an automatically determined threshold were retained. Data acquired for each focus included its average radiance [in photons/s/cm^2^/steradian (sr)], its surface area (in cm^2^), and its photon flux (in photons/s), the latter being equal to the product of the average radiance, the surface area, and 4π. For each anatomical region, the number of foci was determined by overlaying bioluminescent and photographic images, and average radiance and average surface area of foci by determining the arithmetic mean of the radiance and surface area of individual foci. Total photon flux (in photons/s) in each anatomical region was determined by summing the photon flux for individual foci observed within the region.

### Evaluation of transgene expression by reverse transcription quantitative PCR

Ad5-*ova* was adjusted to 2.5 × 10^9^ TCID_50_/ml in PBS and a 200 μl volume (5 × 10^8^ TCID_50_) was administered to fasted mice (three per strain) by the intragastric route as described above. Twenty-four hours later, the mice were euthanized and organs—intestine, MLNs, spleen, and a small lobe of liver—collected. Peyer's patches, identified by visual observation, were excised and reserved. Intestinal contents were removed by flushing with ice-cold PBS. Short sections of duodenum, jejunum, and ileum, as well as the entire cecum, were collected from the intestine. Intestinal and extra-intestinal tissue samples were stored in RNAlater stabilization solution (Ambion) at −20°C.

For isolation of RNA, tissue samples—15–20 mg or entire Peyer's patches and MLNs—were minced and disrupted in 0.6 ml of lysis buffer (RLT solution, Qiagen) containing 1% (v/v) β-mercaptoethanol (β-ME), by using a tissue homogenizer and ceramic lysing beads (Fast Prep-24 instrument and Lysing matrix D, both of MP Biomedicals). The lysate was clarified by centrifugation for 3 min at 10,000 × g, and total RNA was isolated from the supernatant by using commercially available materials (RNeasy Mini Kit, Qiagen) according to the recommendations of the manufacturer. Residual DNA was removed by on-column DNase digestion using the RNase-Free DNase Set (Qiagen). The absorbance of the RNA samples was measured at 260 and 230 nm, to provide an estimation of quantity (260 nm) and purity (260/230 nm) (NanoDrop 2000, Thermo Scientific).

Transgene-specific (*ova*) mRNA was quantified by reverse transcription quantitative PCR (RT-qPCR) by using commercially available reagents (SuperScript®One-Step RT-PCR System with Platinum®Taq DNA polymerase, Invitrogen) and a Light cycler 96 instrument (Roche). Primers and TaqMan probes specific for *ova* and murine β-actin were purchased from ThermoFisher Scientific (Taqman Gene Expression Assays FAM-MGB Gg03366804_m1 and VIC-MGB Mm00607939_s1, respectively). *Ova* and β-actin mRNA were amplified in separate reactions, since preliminary experiments had shown that the sensitivity of detection of *ova* transcripts was diminished in a multiplex context. Relative gene expression was determined by the 2^−ΔΔC_T_^ method (Livak and Schmittgen, 2001). Briefly, the threshold cycle (C_t_) for the *ova* target gene was normalized to that of the β-actin internal control gene and then expressed relative to that of a calibrator tissue, corresponding to the tissue for which the lowest level of *ova* mRNA was detected. The quantity of *ova* mRNA was thus expressed as fold difference as regards that of the common calibrator tissue.

### Simultaneous detection of *Luc* mRNA and enzymatic activity

Ad5-*luc* was adjusted to 2.5 × 10^9^ TCID_50_/ml in PBS and a 200 μl volume (5 × 10^8^ TCID_50_) was administered to a group of five fasted mice by the intragastric route as described above. Twenty-four hours later, tissue samples—~50 mg of distal ileum and apex cecum and entire Peyer's patches—were collected as described above and maintained in culture medium on ice. Peyer's patches were pooled for each mouse. Tissue samples were minced (except Peyer's patches) and then suspended in 600 μl of lysis buffer [25 mM Tris pH 7.8, 8 mM MgCl_2_, 0.1% Triton X-100 (v/v), 15% glycerol (v/v)] and immediately lysed as described for quantification of ova mRNA.

To recover RNA, a 300 μl volume of lysate was combined with 600 μl of lysis buffer (with β-ME) for RNA extraction (RNeasy Mini Kit, Qiagen) and frozen at −20°C until thawed for extraction of RNA as previously described for quantification of *ova* mRNA. Transgene-specific mRNA (*luc*) was quantified by RT-qPCR, as described for *ova* mRNA but with primers and TaqMan probe specific for *luc* (Taqman Gene Expression Assay FAM-MGB Mr03987587_mr, Thermo Fisher Scientific). Relative gene expression was determined by the 2^−ΔΔC_T_^ method as described for *ova* mRNA. To quantify luc activity, the remaining lysate (~300 μl) was maintained on ice for 30 min. The lysate was transferred to a fresh centrifuge tube and clarified by centrifugation for 3 min at 10,000 × g. Supernatant was stored at −20°C until analysis. Protein concentration and luc activity were determined by using commercially available reagents (Pierce BCA Protein Assay Kit, Thermo Fisher Scientific and Bright-Glo Luciferase Assay System, Promega, respectively), and for the latter, by using the IVIS 200 imaging system (PerkinElmer).

### Localization of transgene expression by immunohistochemistry

Twenty-four hours after intragastric administration of Ad5-*luc* or co-administration of Ad5-*luc* and Ad5-*gfp* vectors, intestines were removed and thoroughly rinsed as described above. Tissue samples—apex cecum and portions of the distal ileum comprising Peyer's patches—were collected and immediately fixed in PBS-4% paraformaldehyde for 2 h at room temperature. Tissues were pre-embedded by incubation in PBS containing 12% sucrose for 3 h at 4°C, followed by overnight incubation in PBS containing 20% sucrose at 4°C. The tissues were then embedded in Tissue-Tek optimal cutting temperature compound (O.C.T.) and snap-frozen in isopentane chilled in the vapor phase of liquid nitrogen. Frozen tissue sections (7 μm) were prepared by using a cryostat (Leica CM3050 S) and deposited on slides (Superfrost Ultra Plus, Thermo Scientific). Slides were stored at −20°C until use.

For immunolabeling, slides were washed for 5 to 10 min in PBS to remove O.C.T. Sections were encircled by using a hydrophobic marker and then blocked with PBS containing 5% goat serum and 2% BSA for 15 min at room temperature (100 μl/section). After rinsing with PBS, the sections were labeled overnight at 4°C with rabbit serum directed against firefly luc (Abcam, ab21176), diluted to 1/400 in PBS 0.3% BSA (10 μg/ml final; 50 μl/section). As a negative control, certain sections were incubated with non-immune rabbit serum diluted in the same manner. After three washes in PBS, endogenous biotin was blocked by using commercially available reagents (Endogenous biotin-blocking kit, Molecular Probes-Life Technologies, E-21390), according to the manufacturer's recommendations. After three washes with PBS, the sections were incubated for 1 h at room temperature with a biotin-conjugated secondary antibody directed against rabbit IgG (Biotin-Mouse anti-rabbit IgG (H + L), Thermo Scientific # 31824), diluted 1/100 in PBS 0.3% BSA (50 μl/section). After three washes with PBS, the sections were incubated for 30 min at room temperature with Alexa Fluor 555-conjugated streptavidin (Life Technologies, S32355) diluted 1/1000 (2 μg/ml final) in PBS 0.3% BSA (50 μl/section). After three washes in PBS, the sections were labeled with 4′,6-diamidino-2-phenylindole (DAPI) (1 μg/ml final; 50 μl/section) for 10 min at room temperature. After two washes, one with PBS and the second with water, the sections were mounted in Immu-Mount medium (Thermo Scientific) for observation under fluorescence microscopy.

### Fluorescent labeling of HAdV-5

Particles of Ad5-*luc* or Ad5-*ova* were labeled with the fluorescent probe Alexa Fluor 488 by using Alexa Fluor 488 5-SulfodichloroDP ester (Invitrogen), according to the manufacturer's recommendations. Briefly, 50 μl of a 1 mM solution of Alexa Fluor 4885-SDP ester in DMSO was prepared to which a 0.5 ml volume of a viral suspension containing 10^12^ physical particles of a HAdV-5 vector in 1 M sodium bicarbonate buffer (pH 8.5) was immediately added. After 1 h at room temperature, labeled virus (Alexa488-Ad5) was separated from free dye using a gel filtration column (ZebaTM desalt spin columns, Pierce Biotechnology).

### Tracking fluorescent-labeled HAdV-5 in ligated intestinal loops

Ligated intestinal loops were prepared in BALB/c mice as previously described (Rey et al., 2004). Briefly, Alexa488-Ad5-*ova* was adjusted to 1 × 10^12^ physical particles/ml and a 100 μl volume (1 × 10^11^ PP or ~1 × 10^8^ TCID_50_) injected into the lumen of the ligated loop using a 1 ml U-100 insulin syringe. After 1 or 4 h, mice were sacrificed and the ligated intestinal loop was recovered. After extensive washing with 20 ml of ice-cold PBS, the loop was fixed by immersion in 1 ml of PBS-4% paraformaldehyde for 2 h at room temperature. The tissue was pre-embedded, embedded in O.C.T. compound and snap-frozen, as described above. Frozen sections (7 μm) were prepared as described above. Sections were washed two times for 10 min in 200 ml of PBS at room temperature. Slides were stored at −20°C until thawed for staining.

Immunolabeling was performed in the dark to preserve fluorescence. Slides were washed for 15-20 min in PBS with mild rotation to remove O.C.T. and then blocked using 5% FCS for 15 min. After a single wash in PBS, sections were blocked using commercial Fc block CD16/32 (BD Biosciences) at 1/300 dilution for ~10 min. Slides were then washed once in a large volume of PBS and allowed to dry before labeling. For labeling DCs, biotin-conjugated anti-CD11c mAb (clone HL3, BD Biosciences) was applied at a 1/10 dilution overnight at 4°C. Slides were washed briefly without agitation and incubated with the secondary reagent, streptavidin-Cy5 (Amersham), at 1/500 dilution for 30 min. Nuclei were stained with DAPI at the same time as the secondary reagent using commercial DAPI stain (InVitrogen) at 1/300 dilution. After staining the slides were washed as described earlier and dried. On selected sections, M-cell staining was performed using rhodamine-labeled *Ulex europaeus* agglutinin-1 (UEA-1) (Vector Laboratories) at 1/50 dilution. Finally, VectaShield was added to sections before applying cover slips. The slides were visualized by laser scanning confocal microscope (Carl ZEISS; LSM 710) using Zen 2009 imaging software.

### Tracking fluorescent-labeled HAdV-5 after intragastric administration

Alexa488-Ad5-*luc* was adjusted to 1 × 10^12^ physical particles/ml in PBS and a 200 μl volume (2 × 10^11^ PP or ~1 × 10^9^ TCID_50_) was administered to fasted C57Bl/6 and BALB/c mice by the intragastric route as described above. Twenty-four hours later, mice were euthanized and luc expression was evaluated in intact intestinal tissues by bioluminescence imaging as described above. For mice displaying visible bioluminescent foci, the apex cecum, and Peyer's patches were collected and prepared for cryosecting. For immunolabeling, sections were blocked and endogenous biotin quenched, as described for localization of transgene expression. Sections were then labeled overnight at 4°C with biotin-conjugated anti-mouse CD11c (clone HL3, BD Biosciences) and rabbit anti-human lysozyme (Dako), diluted 1/10 and 1/200, respectively, in PBS 0.3% BSA (50 μl/section). For negative controls sections were labeled with non-immune rabbit serum (1/400 dilution). After washing three times, sections were incubated with secondary reagents: Alexa Fluor 647-conjugated goat anti-rabbit IgG and Alexa Fluor 555-conjugated streptavidin (both from Molecular probes), diluted 1/200 and 1/1000, respectively, in PBS 0.3% BSA (50 μl/section). After washing three times, sections were stained with DAPI and mounted as described for localization of transgene expression. The slides were visualized by laser scanning confocal microscopy, using a LEICA SP5 instrument equipped with a HCX PL Apo CS 63X NA1.4 objective.

### Statistical analysis

Two-way ANOVA was used to evaluate the impact of anatomic location and mouse strain on total photon flux, number of bioluminescent foci and their average radiance and surface area. Multiple pairwise comparison of means was then performed by using the Tukey method. As regards the impact of anatomic location on quantity of *luc*-specific mRNA and specific activity, medians were first compared by using the Kruskal–Wallis test. When different, these were compared pair-wise by applying the Wilcoxon-Mann-Whitney test and Bonferroni correction for multiple comparisons. Analyses were performed by using R (R Core Team, 2017) or GraphPad Prism software, version 6.0c (GraphPad Inc, San Diego, CA, USA).

## Results

### Intramuscular but not intragastric administration of Ad5-*Ova elicits* circulating ova-specific antibodies in mice

**Figure 1:**
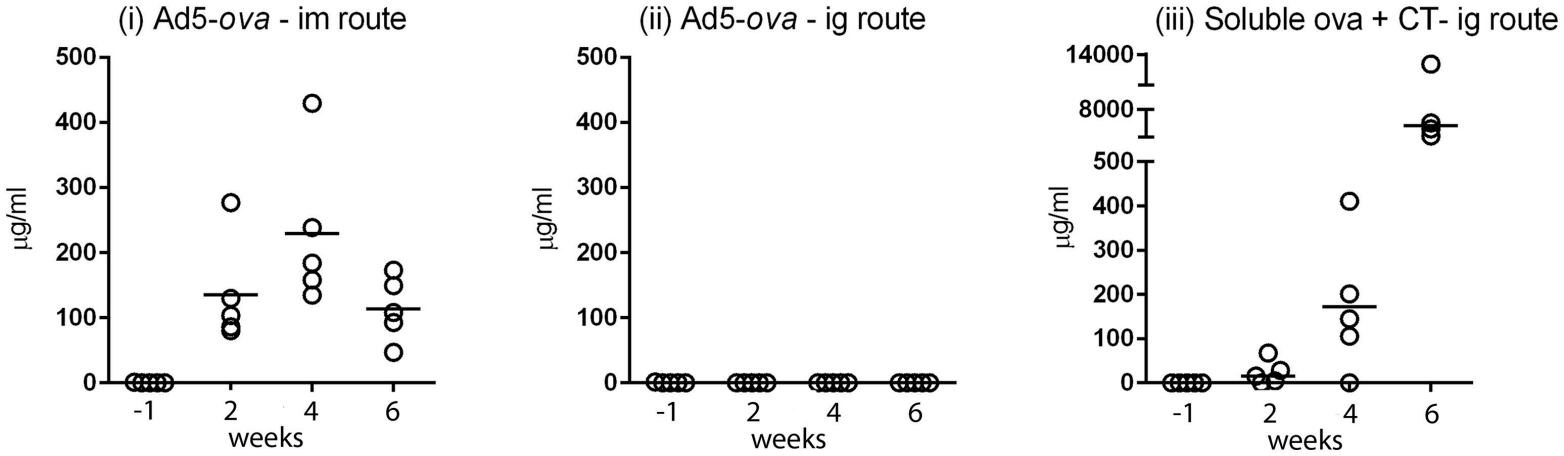
Comparison of humoral responses elicited after intramuscular and intragastric administration of an adenovirus-based vector in mice. A non-replicative HAdV-5-based vector expressing chicken ovalbumin (Ad5-*ova*) was administered to groups of five C57Bl/6 mice at day 0 and 28 by the intramuscular **(i)** or intragastric **(ii)** route, and, as a positive control, soluble ova with CT was administered at day 0, 7, 14, and 28 by the intragastric route **(iii)**. Anti-ova Ig in sera was measured by ELISA on immobilized ova. Responses were determined in reference to a standard curve constructed for a mouse mAb directed against ova, and expressed as concentration of mAb. Data for the high dose of Ad5-*ova* (5 × 10^8^ TCID_50_) are shown in **(i,ii)**.

The efficiency with which Ad5-*ova* elicits antibodies (including IgG, IgM, and IgA) directed against ova was compared in mice after intragastric (ig) and intramuscular (im) administration (Figure 1). After im delivery of 5 × 10^8^ TCID_50_ of Ad5-*ova*, all mice had readily detectable titers of anti-ova antibodies in serum as early as 2 weeks after the first administration and at all subsequent time points (Figure 1i), whereas anti-ova antibodies were not detected in any of the mice at any time after ig delivery of the same dose (Figure 1ii). A robust anti-ova humoral response was nonetheless obtained in control mice after ig administration of soluble ova in the presence of the CT adjuvant (Figure 1iii). Of note, while this last regimen elicited a measurable pan-Ig (IgG, IgM, and IgA) response against ova in the intestinal compartment, as measured in fecal extracts, administration of Ad5-*ova*, irrespective of delivery route, did not (data not shown). Induction of a systemic humoral response by non-replicative HAdV-5-based vaccines was thus vastly less effective by the ig than im delivery route as, similar to previous observations made in mice for other transgene-encoded antigens (Vos et al., 2001; Xiang Z. et al., 2003). Indeed, im administration of even 5 × 10^6^ TCID_50_ of Ad5-*ova*, a 100-fold lower dose, sufficed for induction of a robust humoral immune response directed against the transgene product (data not shown).

### After intragastric administration of Ad5-*luc*, the level of transgene expression peaks after ~24 h

**Figure 2:**
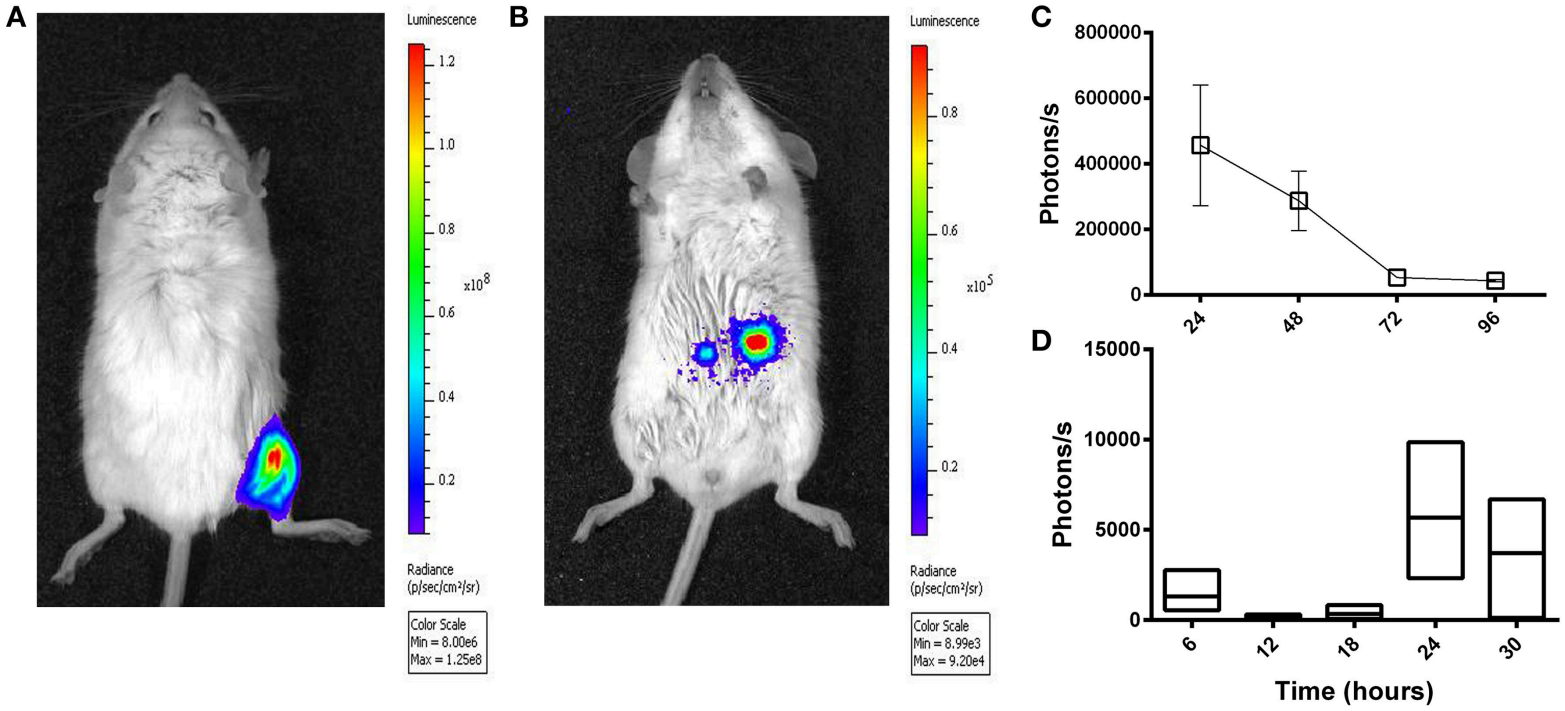
Temporal transgene expression after intragastric administration of an adenovirus-based vector in mice, as revealed by bioluminescence imaging. A non-replicative HAdV-5-based vector (Ad5-*luc*) expressing firefly luciferase was administered (5 × 10^8^ TCID_50_) to mice by intramuscular **(A)** and intragastric **(B–D)** routes. Twenty-four hours later or at different time points thereafter for intramuscular and intragastric administration, respectively, anesthetized mice received d-luciferin by the intraperitoneal route. Approximately 5 min later, photon emission was detected by means of the IVIS 200 imaging system (Xenogen) for live BALB/c mice **(A–C)**, or following euthanasia and rapid dissection of mice, for the entire intestinal tract of C57Bl/6 mice **(D)**. **(A)** Bioluminescence was analyzed by using Living Image software and expressed as radiance in photons/second/cm^2^/steradian **(A,B)** or photon flux in photons/second **(C,D)**, for a single user-defined abdominal zone in **(C)** or as the sum of automatically drawn bioluminescent foci in **(D)**. The representative BALB/c mouse shown in **(B)** received the vector 24 h earlier. Arithmetic means ± SEM for pairs of mice (except the 72 h time point concerning a single mouse) are shown in **(C)**; Boxes in **(D)** depict arithmetic means with maximum and minimum values for groups of three mice.

A HAdV-5-based vector (Ad5-*luc*) encoding firefly luciferase (luc) was delivered to BALB/c mice by the ig route and transgene expression was evaluated at different time points thereafter by *in vivo* bioluminescence imaging. Though transgene expression was readily observed in the hind limb after im delivery of the same quantity of vector (Figure 2A), the magnitude of bioluminescence observed in the abdominal region after ig administration was inferior by approximately three logs (Figure 2B). The highest values were obtained at the first time point examined, 24 h (Figure 2C). Earlier time points were next examined after ig delivery of Ad5-*luc* to C57Bl/6 mice, and after euthanasia and dissection, bioluminescence could be observed in the abdominal cavity at 24 and 30 h time points (Figure 2D). In view of these results, the 24 h time point was selected for all subsequent analyses of transgene expression by bioluminescence imaging.

### After intragastric administration of Ad5-*Luc*, transgene expression–as determined by bioluminescence imaging—is confined to the intestine but varies according to the anatomical region

**Figure 3:**
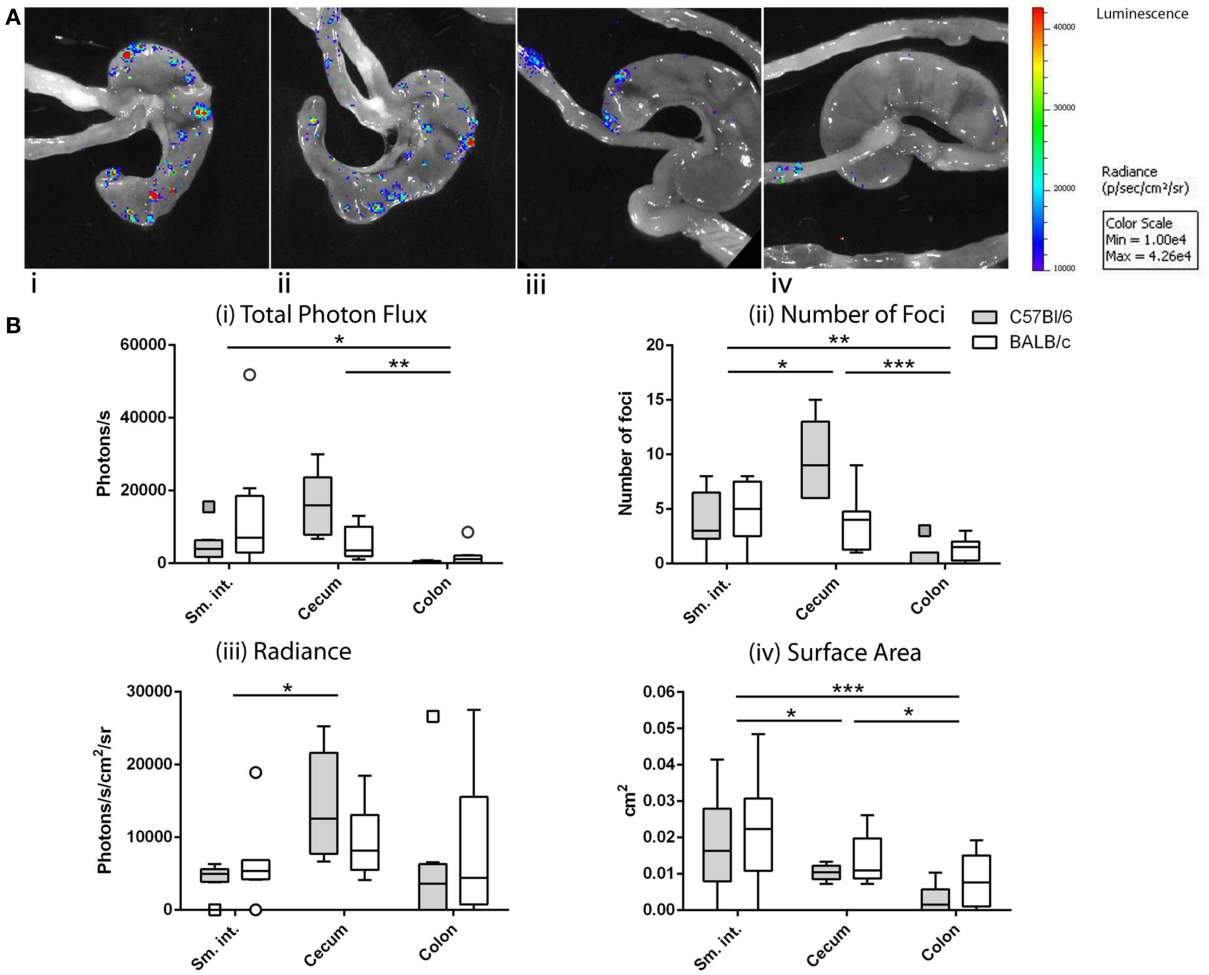
Anatomy of transgene expression after intragastric administration of an adenovirus-based vector in mice, as revealed by bioluminescence imaging. As described in the legend of Figure 2, mice received Ad5-*luc* (5 × 10^8^ TCID_50_) by the intragastric route and d-luciferin 24 h later. Mice were euthanized 5 min later and rapidly dissected to display the entire intestine. Photon emission was detected as described in the legend of Figure 2. **(A)** Magnification of the resulting images reveals multiple bioluminescent foci in cecum and ileum of C57Bl/6 **(i,ii)** or BALB/c **(iii,iv)** mice. **(B)** Foci of C57Bl/6 and BALB/c mice were analyzed by using Living Image software and attributed to various intestinal tissues (small intestine, cecum, and colon). Data are expressed for individual tissues as total photon flux (sum of the photon flux of individual bioluminescent foci) **(i)**, number of foci **(ii)**, average radiance of foci **(iii)**, and average surface area of foci **(iv)**. Boxes of box and whisker plots show 25, 50, and 75th percentiles. Whiskers are drawn according to the Tukey method. For each mouse strain, data from two independent experiments involving four mice/experiment have been pooled. Arithmetic means were compared by the Tukey method: ^*^P < 0.05; ^**^P < 0.01; ^***^P < 0.001.

After ig inoculation of Ad5-*luc*, C57Bl/6, and BALB/c mice were dissected to expose the viscera for bioluminescence imaging. Transgene expression was never observed in extra-intestinal tissues such as liver and spleen, and not even in the mesenteric lymph nodes (MLNs) to which intestinal tissues drain (data not shown). Bioluminescence was, however, observed throughout the intestinal tract—and more particularly within the distal small intestine (ileum), the cecum, and proximal colon—taking the form of multiple small foci (Figure 3A).

For the purpose of analysis, we made the assumption that the bioluminescent foci corresponded to instances of translocation and transduction of intestinal target cells and that photon flux was related to the level of transgene expression. We further assumed that the number of foci reflected the number of productive—that is, leading to transduction—crossings, that the radiance of foci was related to the density of transduced cells and/or their level of transgene expression and, finally, that the surface area was related to the extent of the transduced zone. Based on these considerations, the total photon flux, number of foci, radiance, and surface area were compared for three anatomical zones—namely, the small intestine, cecum and colon—for C57Bl/6 and BALB/c mice (Figure 3B) mice.

Comparison of these four variables by two-way ANOVA revealed statistically significant variation according to anatomic location but not to strain of mouse. Total photon flux was higher in the cecum and small intestine than in the colon (Figure 3Bi). The number of foci was also greater in the cecum and small intestine than in the colon, with greater numbers found in the cecum than in the small intestine (Figure 3Bii). The radiance of foci was also higher in the cecum than in the small intestine, but with no discernible difference between these tissues and the colon (Figure 3Biii). Regarding the surface area, the foci of both cecum and small intestine were significantly larger than those found in the colon, though those of the small intestine were larger than those of the cecum (Figure 3Biv).

Taken together, the total photon flux—and thus presumably the magnitude of transgene expression—was similarly high in the small intestine and cecum, the crossing events (number of foci) in the cecum being more frequent and leading to a greater intensity of transgene expression (radiance), while those in the small intestine giving rise to more extensive zones of transduction (surface area).

### After intragastric administration of Ad5-*Ova*, transgene expression—as determined by RT-qPCR—is confined to the intestine but varies according to the anatomical region

**Figure 4:**
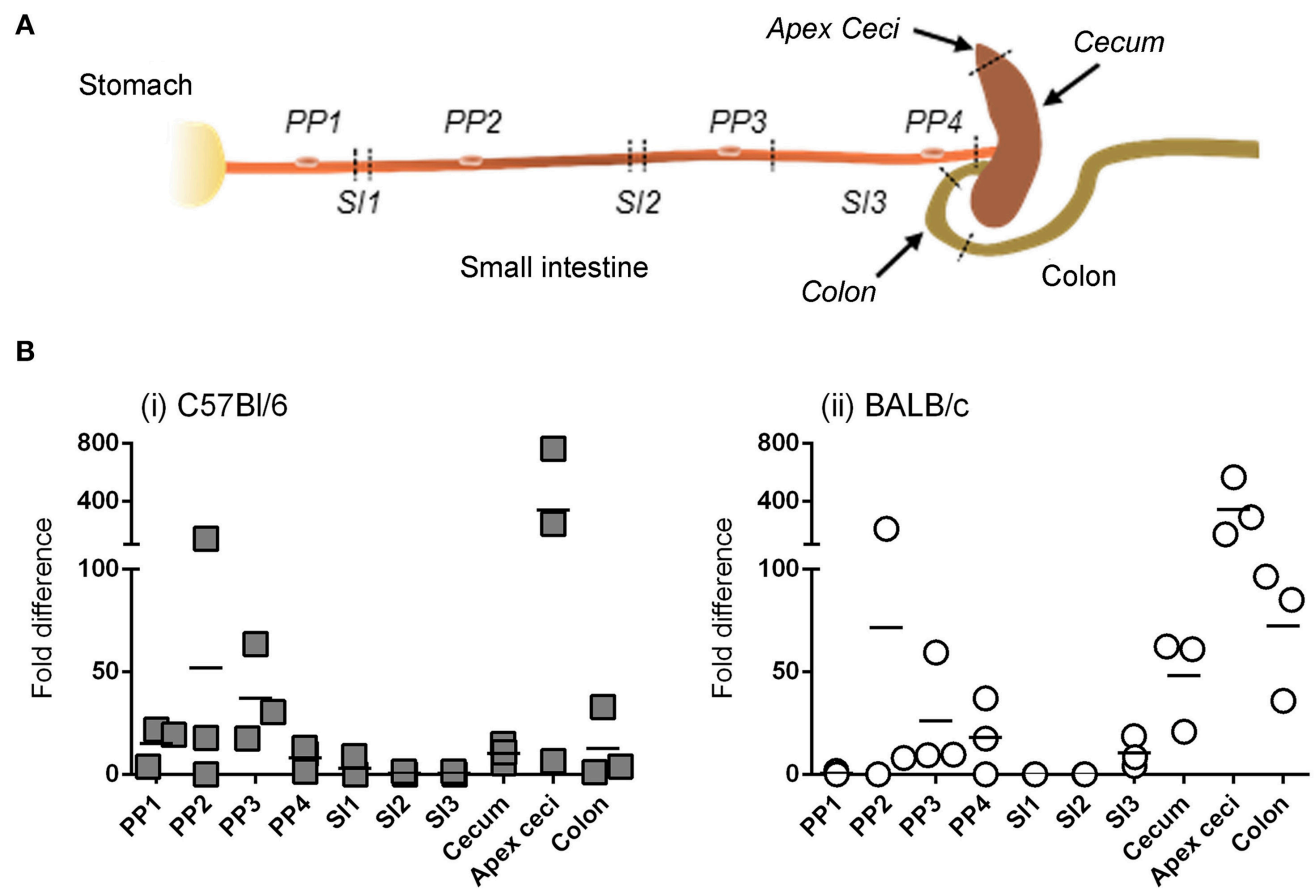
Anatomy of transgene expression after intragastric administration of an adenovirus-based vector in mice, as revealed by RT-qPCR. A non-replicative HAdV-5-based vector (Ad5-*ova*) expressing ova was administered (5 × 10^8^ TCID_50_) to mice (three per strain) by the intragastric route. Twenty-four hours later mice were euthanized and tissues were collected from different anatomical zones of the intestine (sampling sites indicated by italicized text) as well as from extra-intestinal organs (mesenteric lymph nodes, spleen, liver) **(A)**. **(B)** Total RNA was extracted from tissue and ova and β-actin transcripts were quantified by RT-qPCR for C57Bl/6 **(i)** and BALB/c **(ii)** mice. The relative expression level of ova RNA was determined by the 2^ΔΔC_T_^ method; that is, after normalization against β-actin transcripts and relative to that of a positive internal reference tissue, and expressed as relative units. Arithmetic means are indicated.

Detection of bioluminescence requires not only sufficient expression of transgene-encoded luc, but also sufficient diffusion of the d-luciferin substrate and adequate concentrations of the enzymatic co-factors of luc; that is, O_2_ and ATP. We therefore studied the anatomy of transgene expression by a second technique that was free from these constraints. In particular, after ig delivery of Ad5-*ova* to C57Bl/6 and BALB/c mice, *ova*-specific mRNA was quantified by RT-qPCR in tissues collected throughout the intestine (Figure 4A), as well as in extra-intestinal tissues.

Similar to results obtained when transgene expression was tracked by bioluminescence imaging, detectable levels of *ova* mRNA were not observed in extra-intestinal tissues, even in the MLNs that drain intestinal tissue, at 24 and 48 h (data not shown). For both C57Bl/6 (Figure 4Bi) and BALB/c (Figure 4Bii) mice, however, *ova* transcripts were detected throughout the intestinal tract, including not only the cecum (and more particularly the blind end of the cecum, or “apex ceci”) and colon, but also Peyer's patches, to which bioluminescent foci had rarely been attributed. Moreover, relatively little transgene expression was detected in the segments of the small intestine examined, although this tissue had appeared to be a major site of transgene expression when appreciated by bioluminescence imaging (Figure 3B).

### After intragastric administration of Ad5-*luc*, the relationship between the magnitude of bioluminescence and the quantity of *luc* mRNA varies according to the anatomical region

**Figure 5:**
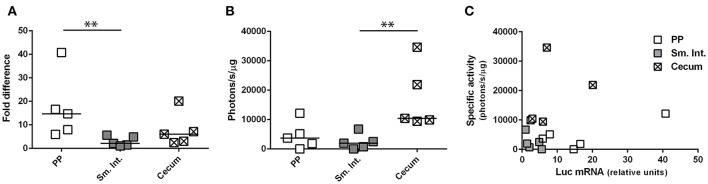
Concurrent evaluation of transgene-specific enzymatic activity and mRNA expression level. Ad5-*luc*, described in the legend to Figure 1, was administered (1 × 10^9^ TCID_50_) to C57Bl/6 mice by the intragastric route. Twenty-four hours later mice were euthanized and tissues–including Peyer's patches, distal small intestine and cecum–were collected. Following tissue disruption, homogenates were divided for extraction of total RNA and quantification of luc transcripts **(A)** or analysis of luc activity by luminometry **(B)**. **(A)** The relative expression level of luc RNA was determined by the 2^ΔΔC_T_^ method, after normalization against β-actin transcripts and relative to that of a positive internal reference tissue, and expressed as relative units. **(B)** Luciferase activity was normalized to protein concentration and expressed as specific activity (photons/s/μg total protein). Medians are indicated in **(A,B)**. **(C)** Bioluminescence is plotted for individual tissues as a function of luc mRNA level. ^**^P < 0.01; as determined by the Wilcoxon-Mann-Whitney test.

Depending upon the gene product (mRNA or protein) used to appreciate transgene expression—bioluminescence imaging or RT-qPCR—the relative levels of transgene expression in different tissues appeared to be different; that is, relatively high levels of expression in small intestine by bioluminescence imaging but in Peyer's patches by RT-qPCR. To determine whether this difference was related to the use of HAdV-5-based vectors expressing different transgenes (*ova* or *luc*) we performed an additional experiment in which a single vector (Ad5-*luc*) was administered by the ig route to C57Bl/6 mice. After harvesting tissues—Peyer's patches, distal small intestine and cecum—the lysates were split for assay of bioluminescence by luminometry and *luc* transcripts by RT-qPCR. We observed that *luc* transcripts were detectable in all three tissues, with relatively higher levels being found in Peyer's patch than in small intestine (Figure 5A), in keeping with observations made for ova mRNA (Figure 4B). As regards bioluminescence, however, higher photon flux, as normalized to protein concentration, was observed in the cecum than in the small intestine (Figure 5B). To capture the relationship between levels of *luc* mRNA and bioluminescence in the different tissues, specific activity (photons/s/μg total protein) was plotted as a function of relative levels of *luc* transcripts (Figure 5C). Generally speaking, relatively low levels of *luc* mRNA in cecum gave rise to relatively high levels of bioluminescence, while relatively high levels of *luc* mRNA in Peyer's patches gave rise to relatively low levels of bioluminescence. These observations suggest that post-transcriptional regulation of transgene expression might differ between Peyer's patches and cecum. In this particular experiment, both *luc* mRNA and bioluminescence were low for the small intestine, possibly because the tissue samples collected for analysis did not coincide with highly transduced zones.

### After intragastric administration of HAdV-5-based vectors, transgene-encoded protein is produced in the intestinal epithelium

**Figure 6:**
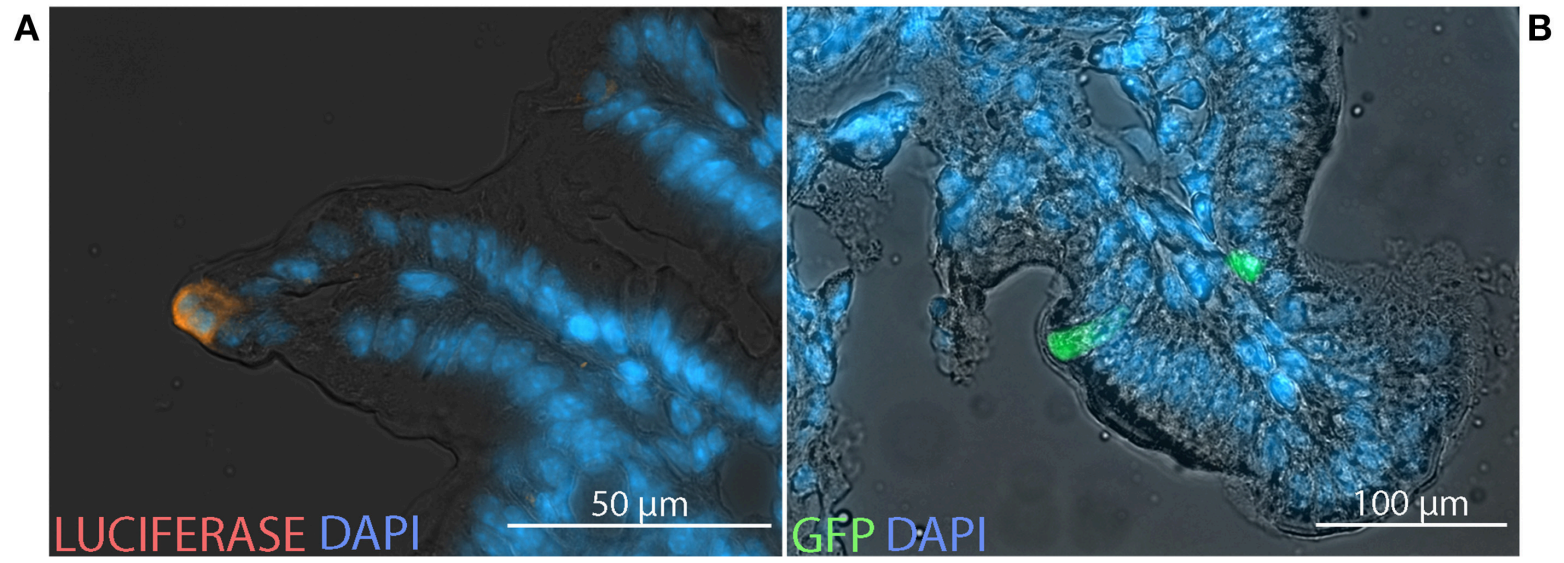
Transgene expression at the cellular scale. Non-replicative HAdV-5-based vectors expressing firefly luciferase (Ad5-*luc*) or green fluorescent protein (Ad5-*gfp*), were co-administered (5 × 10^8^ TCID_50_ of each) to mice by the intragastric route. Following bioluminescence imaging—performed as described in the legend of Figure 1—bioluminescent zones of cecum and distal small intestine were collected from C57Bl/6 **(A)** and BALB/c **(B)** mice, respectively, and processed for fluorescence microscopy. Cryosections were stained with antibodies directed against luciferase (orange) and 4′,6-diamidino-2-phenylindole (DAPI; blue).

To characterize the cells in which transgene-encoded protein is expressed, equal doses of two HAdV-5-based vectors, expressing luc or GFP, were co-administered by the ig route to C57Bl/6 and BALB/c mice. After immunolabeling with antibodies directed against luc and analysis by fluorescence microscopy, expression of *luc* and GFP was observed within cells located in the epithelium of both cecum (Figure 6A) and distal small intestine (Figure 6B).

### Selective binding of fluorescent-labeled HAdV-5 to intestinal mucosa and translocation of Alexa488-Ad5 across intestinal mucosa via m-cells after 1 h

**Figure 7:**
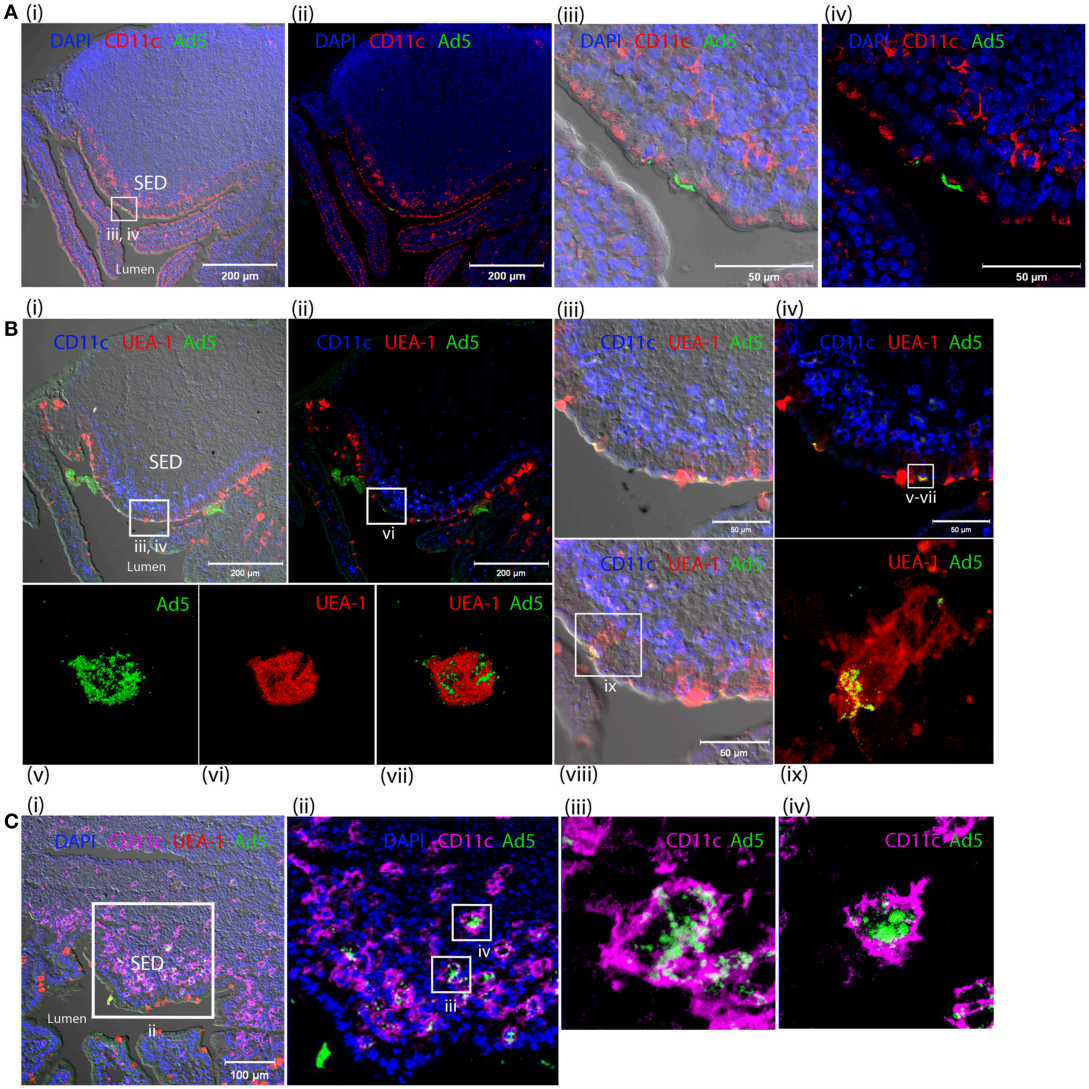
Interaction of HAdV-5 viral particles with the intestinal mucosa. Fluorescent capsid-labeled HAdV-5 particles (1 × 10^11^ physical particles) were inoculated into ligated intestinal loops comprising a Peyer's patch. Tissue was collected after 1 **(A,B)** or 4 h **(C)** and processed for immunohistochemistry, prior to analysis by laser scanning confocal microscopy. **(A)** View of the entire Peyer's patch with **(i)** and without **(ii)** Nomarski, and zoom of selected area of Ai with **(iii)** and without **(iv)** Nomarski. Labeled HAd5 (green) and staining for CD11c (magenta) and DAPI (blue) are shown. **(B)** View of the entire Peyer's patch in a neighboring section with **(i)** and without **(ii)** Nomarski, and zoom (4×) of selected area of (Bi) with **(iii)** and without **(iv)** Nomarski. **(v–vii)** 3D acquisition of an M cell indicated in **(iv)**. **(viii)** View of a portion of the FAE on a section neighboring that shown in (Bi). **(ix)** Zoom on M cell indicated in **(viii)**. Labeled HAdV-5 (green) and staining for UEA-1 (red) and CD11c (blue) are shown. **(C) (i)** View of the entire SED with Nomarski **(i)**. **(ii)** Zoom of selected area of Ci. **(iii,iv)** 3D acquisition of two CD11c^+^ cells from **(Cii)**. Labeled HAdV-5 (green) and staining for UEA-1 (red in **i,ii**) and CD11c (magenta) are shown.

One hour after inoculation of Alexa488-labeled HAdV-5 (Alexa488-Ad5) into ligated intestinal loops, the latter were immuno-stained for the macrophage and DC marker CD11c and analyzed by laser scanning confocal microscopy (Figures 7Ai–iv). Alexa488-Ad5 was observed to be concentrated at discrete sites at the apical surface of the FAE, but to be absent from the SED region (Figures 7Ai,ii). Thus, HAdV-5 appeared to interact with particular cells in the Peyer's patch epithelium at the 1 h time point.

To characterize binding of Alexa488-Ad5 to the FAE, tissue sections were stained with anti-CD11c and, to visualize M cells, with the UEA-1 lectin microscopy (Figures 7Bi–ix). Image analysis revealed that Alexa488-Ad5 and UEA-1 co-localized, suggesting that Alexa488-Ad5 had selectively adhered to M cells (Figures 7Biii,iv) overlaying the SED region. Moreover, higher magnification revealed that Alexa488-Ad5 was internalized by M cells (Figures 7Bv–vii), most likely as intact particles displaying preserved fluorescence. Consistent with the capacity of M cells to transport large antigens including viruses and bacteria in the absence of degradative processes, the 3D acquisition on one M cell showed a virus being released at the basolateral surface (Figure 7Bix), suggesting that at the 1 h time point, Alexa488-Ad5 is available to immune cells present in the SED region of the FAE.

### Capture of fluorescent-labeled HAdV-5 by peyer's patch phagocytes in SED of intestinal mucosa after 4 h

Four hours after inoculation of Alexa488-Ad5 in the ileal loop, imaging revealed that Alexa488-Ad5 had been captured by CD11c^+^ cells located in the SED region of intestinal mucosa (Figures 7Ci,ii). Little virus was observed in association with other cells that did not express the CD11c marker. The 3D acquisition of individual CD11c^+^ cells revealed that the virus had been internalized (Figures 7C, iii,iv), with the intensity of intracellular staining suggesting that viral particles had accumulated in particular intracellular compartments.

### Capture of fluorescent-labeled HAdV-5 by lysozyme-positive peyer's patch phagocytes of intestinal mucosa after 24 h

**Figure 8:**
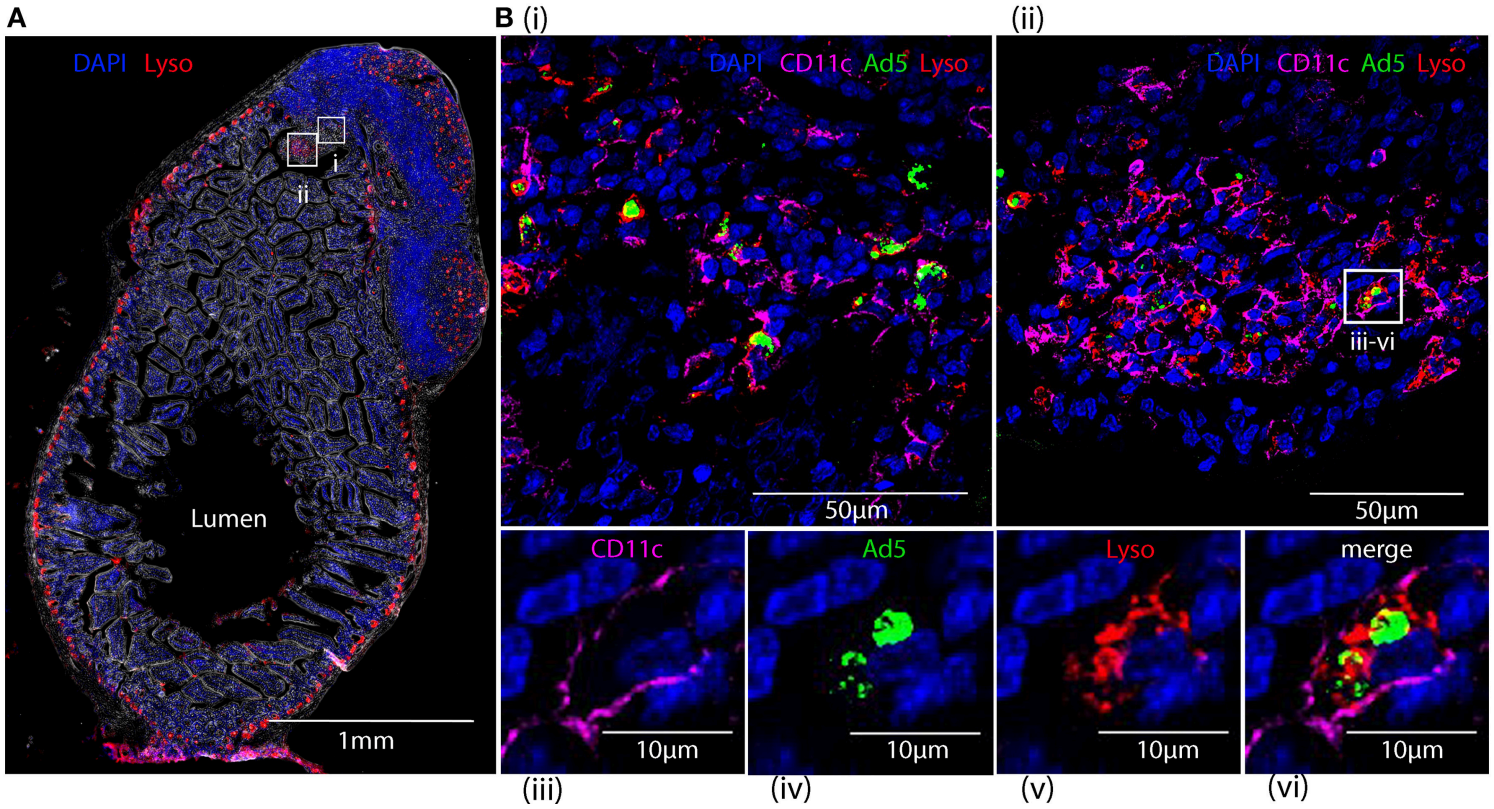
Tracking HAdV-5 after intragastric administration. Fluorescent capsid-labeled HAdV-5 particles (2 × 10^11^ physical particles) were administered to C57Bl/6 mice by the intragastric route. Twenty-four hours later mice were euthanized and tissues were collected and processed for immunohistochemistry, prior to analysis by fluorescent microscopy **(A)** and laser scanning confocal microscopy **(B)**. **(A)** View of the entire intestinal section including a Peyer's patch with phase contrast. DAPI (blue) and lysozyme (red) are shown. **(B)** Zoom of selected areas of **A(i,ii)** and zoom on a representative cell from **B(ii)** that has captured fluorescent-labeled HAdV-5 particles **(iii–vi)**. Fluorescent-labeled HAdV-5 (green) and staining for DAPI (blue), lysozyme (red) and CD11c (magenta) are shown.

Cryosections of apex ceci and Peyer's patches, collected 24 h after ig administration of Alexa488-Ad5, were labeled with antibodies specific for CD11c and lysozyme, expression of the latter reported to distinguish the largest subset of highly phagocytic DCs in murine Peyer's patches (Lelouard et al., 2010). Imaging revealed that Alexa488-Ad5 had been captured within the Peyer's patch (Figure 8A) by numerous cells (Figures 8Bi,ii). Most, if not all, of the cells bearing Alexa488-Ad5, or fluorescent structural proteins thereof, expressed both CD11c and lysozyme (Figures 8Biii–vi).

## Discussion

To acquire a better understanding of the fate of viral-vectored vaccines in the intestinal milieu, we tracked HAdV-5-based vectors expressing reporter proteins or model antigens at whole body, tissue, and cellular scales after ig administration in mice. At the whole body level, transgene expression—whether evaluated by RT-qPCR or bioluminescence imaging—was not observed in extra-intestinal tissues, such as liver and spleen, although these two organs are highly transduced after parenteral administration of HAdV-5-based vectors. Both lymph nodes and a gut vascular barrier have been described to confine non-invasive luminal bacteria to the intestine (Macpherson and Uhr, 2004; Macpherson et al., 2009; Spadoni et al., 2015), and might possibly restrict access of non-replicative viral particles to lymphatics and blood, respectively.

At the tissue scale, transgene expression—as visualized by bioluminescence imaging—took the form of multiple small bioluminescent foci found primarily in the distal small intestine, cecum and colon. This suggested that the vector crossed the epithelium at multiple sites in different zones of the intestine, whence it was able to transduce cells and produce transgene-encoded protein. The difference in total photon flux among zones was related to variation in the number of bioluminescent foci, as well as to their average radiance and surface area, which we interpret as meaning that the number of productive crossing events and the subsequent level of production of transgene-encoded protein differed in various zones of the intestine. The cecum emerged as a zone of high transgene expression, and may thus represent a major sampling site for HAdV-5 upon ig delivery, and more generally a largely overlooked tissue as regards penetration of luminal viruses and potentially other particulate antigen.

Of note, bioluminescence rarely coincided with visible Peyer's patches or with the functionally related cecal patch, which is the major site of organized lymphoid tissue in the cecum. Given the importance of Peyer's patches in the entry of several enteric viruses and of particulate antigen in general, this result was unexpected. Nevertheless, bioluminescence imaging only permitted observation of crossing events that led to production of enzymatically active luc. Had crossings often been unproductive; that is, not led to uptake by permissive cells and production of transgene-encoded protein, the number of crossing events would have been underestimated. Such indeed appeared to be the case, for when transgene expression was assessed by RT-qPCR, the Peyer's patches emerged as major sites of transgene expression, albeit only at the level of mRNA.

We initially considered it possible that luc had indeed been produced in Peyer's patches, but that bioluminescence had not been observed because local concentrations of enzymatic co-factors (ATP and O_2_) or substrate (luciferin) were insufficient. We were able to rule out this possibility, however, as when co-factors and substrate were provided directly to homogenates of Peyer's patches, specific activity of luc remained low in relation to the quantity of luc transcripts. We thus hypothesize that viral gene expression was subjected to post-transcriptional regulation within at least some cellular targets of HAdV-5 in Peyer's patches. It may be speculated that such regulation is related to robust anti-viral defenses deployed to limit infection in a tissue rendered vulnerable by its antigen sampling activity, or possibly represents a homeostatic mechanism limiting induction of active immunity.

As regards the pathways used to cross the intestinal epithelium, passage of several enteric viruses has been proposed to depend upon Peyer's patches, and more particularly, the M cells of the FAE (Wolf et al., 1981; Gonzalez-Hernandez et al., 2014; Kolawole et al., 2015). This pathway appears to be taken by fluorescent-labeled HAdV-5-based particles as well, as in our study these could be observed to adhere selectively to M cells in ligated intestinal loops. Such particles were rapidly taken up by mononuclear phagocytes within the SED, most or all of which appeared to express lysozyme and thus were likely to correspond to the subset of mononuclear phagocytes expressing lysozyme M (LysoDC) described by Lelouard et al. (2010). These cells may have captured viral particles that had gained entry to the SED after trancytosis by M cells, or may have seized viral particles from within the lumen via transepithelial dendrites before retracting back into the SED (Lelouard et al., 2012).

The dearth of fluorescent particles in other cell types was striking, and posed the question of whether uptake of HAdV-5 particles by LysoDC might be receptor-mediated. If so, it is unlikely that CAR and αv integrins—cell surface-expressed molecules described to be high-affinity attachment (Bergelson et al., 1997) and internalization receptors (Wickham et al., 1993), respectively, for HAd5V—were involved. Indeed, while these molecules are required for high level transduction of highly permissive cell lines, they are unnecessary for transduction of murine DCs (Cheng et al., 2007). Natural IgM antibodies found in sera of mice are known to bind to HAdV-5 particles and promote uptake by Kupffer cells (Qiu et al., 2015). It may be speculated that natural antibodies or other opsonins are able to bind to luminal HAdV-5 and promote adhesion to M cells and/or mononuclear phagocytes of the SED. Although fluorescent vector particles, or structural proteins thereof, were readily detected within mononuclear phagocytes of the SED, strikingly, expression of transgene-encoded protein was not. Similar to conclusions drawn for commensal and pathogenic bacteria (Kadaoui and Corthésy, 2007; Schenk and Mueller, 2007), these cells might constitute yet another firewall against expression of viral genes and hence viral infection. Their role may be analogous to that of Kupffer cells, which sequester systemically delivered adenovirus-based vectors from the bloodstream and limit transgene expression (Khare et al., 2013). Thus, translocation of luminal HAdV-5 into Peyer's patch might represent an impasse in induction of an immune response to transgene-encoded antigen.

In addition to passage across the intestinal epithelium at canonical sites—the FAE of Peyer's patches—our results imply that the vector transduced cells after passage at non-canonical sites. It is of course possible that the bioluminescent foci lying outside prominent Peyer's patches and the cecal patch corresponded to FAE overlying isolated follicles, and that the processes involved in penetration were analogous within and without Peyer's patches. Nevertheless, transgene expression was observed in epithelial cells that did not lie in immediate proximity to lymphoid follicles, suggesting that the vector had in some—and perhaps many—instances transduced cells in the villous epithelium rather than the Peyer's patch. Transgene production in intestinal epithelium has also been noted after introduction of HAdV-5 in the intestinal lumen of rats (Cheng et al., 1997; Foreman et al., 1998). While transduction of epithelial cells could theoretically have been secondary to entry by either apical or basolateral poles, weak expression of CAR and αv integrins at the apical surface of differentiated intestinal epithelial cells is likely to limit entry and transduction of luminal HAdV-5 particles (Walter et al., 1997; Croyle et al., 1998b; Lecollinet et al., 2006). As regards possible transduction at the basolateral pole, luminal vector may have been taken up by dendritic extensions and handed off after retraction through the epithelium. Alternatively, opsonization of vectors may conceivably have promoted interactions with antigen-shuttling receptors at the luminal surface of villous epithelium or with dendritic extensions of phagocytes, and thus enhanced apical or basolateral entry, respectively.

Regarding the higher systemic immune response observed after im administration of Ad5-*ova*, the greater portion of inoculated HAd5-derived vector is confined to the injected muscle and draining lymph nodes thereof (Hemmi et al., 2014). Moreover, the overwhelmingly majority of transgene-encoded protein is produced in the same location; that is, injected muscle and draining lymph nodes (Yang et al., 2003; Kaufman et al., 2010). In the draining lymph nodes, antigen appears to be selectively expressed in conventional DCs (Lindsay et al., 2010; Dicks et al., 2015). Transgene expression is prolonged (Finn et al., 2009), as is presentation of transgene-encoded antigen to antigen-specific CD8^+^ T cells (Yang et al., 2006; Tatsis et al., 2007). Both prolonged transgene expression and antigen presentation by hematopoietic antigen presenting cells are required for optimum priming of the CD8^+^ T cell response (Finn et al., 2009; Bassett et al., 2011). It can be reasonably hypothesized that a key difference between im and ig immunization is that the former supports sufficient transduction of cDCs in secondary lymphoid tissue such that immune responses may be primed, while the latter does not. In keeping with this interpretation, superior immunogenicity of HAdV-5 as compared with human adenoviruses of species E has been associated with substantially higher levels of transgene expression, particularly within draining lymph nodes, after im administration in mice (Dicks et al., 2015).

In conclusion, after ig administration of HAdV-5-based vectors in mice, a large number of crossing events took place throughout the intestine within and without Peyer's patches. Nevertheless, multiple firewalls restricted systemic dissemination of vector and translation of transgene-encoded protein, notably in Peyer's patches. Since the organized lymphoid tissue of Peyer's patches are critical sites for induction of intestinal immune responses, strict repression of translation of viral mRNA after passage via the FAE of Peyer's patches would be expected to restrict immune responses generated to transgene-encoded antigen after oral delivery. Optimization of immune responses elicited by non-replicative versions of HAdV-5, and possibly other viral vectors, is likely to require enhanced production of transgene-encoded protein in the gastrointestinal milieu, potentially by eluding capture by mononuclear phagocytes and promoting transduction of other cell types.

## Author contributions

Conceptualization: BC, JB, and JPR; Formal Analysis: GZ; Investigation: JR, YU, NR, MS, JS, SG, FG, SL, and NC; Resources: MH and SB; Writing—Original Draft: JR and JPR; Writing—Review & Editing: JS, MC, MH, BK, GZ, SB, BC, JB, and JPR; Visualization: JR, NR, and MC; Supervision: NC, NV, SB, BC, JB, and JPR; Project Administration: BC, JB, and JPR; Funding Acquisition: BC, JB, and JPR.

### Conflict of interest statement

The authors declare that the research was conducted in the absence of any commercial or financial relationships that could be construed as a potential conflict of interest.
